# Sub-chronic Hepatotoxicity of *Anacardium occidentale* (Anacardiaceae) Inner Stem Bark Extract in Rats

**DOI:** 10.4103/0250-474X.70482

**Published:** 2010

**Authors:** T. J. N. Okonkwo, O. Okorie, J. M. Okonta, C. J. Okonkwo

**Affiliations:** Pharmaceutical and Medicinal Chemistry Department, University of Port Harcourt, East-West Road, Choba, Port Harcourt, Rivers State, Nigeria; 1 Pharmaceutics /Pharmaceutical Technology Department, Faculty of Pharmaceutical Sciences, University of Port Harcourt, East-West Road, Choba, Port Harcourt, Rivers State, Nigeria; 2 Department of Clinical Pharmacy and Pharmacy Management, Faculty of Pharmaceutical Sciences, University of Nigeria, Nsukka, Enugu State, Nigeria; 3 Biochemistry Division, Department of Chemical Sciences, Novena University, Ogume, Kwale, Delta State, Nigeria

**Keywords:** *Anacardium occidantale*, Anti-nutrients, heavy metals, Liver function indices

## Abstract

The extracts of *Anacardium occidentale* have been used in the management of different cardiovascular disorders in Nigeria. These have necessitated the assessment of the toxicity of this plant extract in sub-chronic administration. The inner stem bark of *Anacardium occidentale* was extracted with 80 % methanol and quantitatively analysed for antinutrients and some heavy metals. The phytochemical compositions and acute toxicity of the extract were determined also. Toxicity profiles of the extract on some liver function parameters were evaluated following a sub-chronic oral administration at doses of 1.44 and 2.87 g/kg. The phytochemical screening of extract revealed the presence of high amount of tannins, moderate saponins and trace of free reducing sugars. The antinutrient levels were 5.75 % (tannins), 2.50 % (oxalates), 2.00 % (saponins), 0.25 % (phytate) and 0.03 % (cyanide). The quantity of iron detected from dried crude was 8.92 mg/100 g, while lead and cadmium were non-detectable. The extract had LD_50_of 2.154g/kg *p.o*. in mice. Sub-chronic administration of the extract significantly increased the serum levels of alanine aminotransaminase and aspartate aminotransaminase, which are indicative of liver damage. The serum levels of alkaline phosphatase and total protein of the treated animals were not significantly increased. The effects of sub-chronically administered extract on hepatocytes were minimal as the serum alkaline phosphatase; total bilirubin and total protein levels in treated animals were not significant (*p*< 0.05). Thus, sub-chronic administrations of *Anacardium occidentale* inner stem bark extract did not significantly (*p*< 0.05) depress the function of hepatocytes in Wistar rats.

The use of medicinal plants in the treatment of ailments in both the developed and developing countries is on the increase. The therapeutic values of most of these herbs are indisputable but their toxicities sometimes limit their clinical uses. Thus the toxicity profile of these herbs must always be considered especially as the doses and dosing regimens of their preparations are not usually determined[1].

*Anacardium occidentale*, commonly known as cashew tree, is a multipurpose tree of the Amazon that grows up to 15 m high. The bark and leaf of the tree are used medicinally, and the fruit has international appeal and market value as food. The shell oil has been authenticated to have medicinal and industrial applications because of its phenol content[2]. In Brazil, cashew fruits and their juice are used to treat fever, sweeten bread and conserve the stomach[2]. In Nigeria, the decoction of root and stem is used as antiinflammatory agent and antidiarrhoea[3].

Cashew fruit is used in the treatment of premature aging of the skin by re-mineralising the skin due to its high vitamin C and mineral contents. Cashew leaf is still widely used in the tropics for the treatment of diarrhoea and colic. In Nigeria, extract of the leaf of cashew has been used to lower blood pressure and sugar[4]. Some tribes of Surinam use the seed oil of cashew as an external wormicide to kill butterfly larva. In Brazil, the tea of the bark is used as douche and as an astringent to stop bleeding after tooth extraction[3]. A wine made from the fruit is used for dysentery in other parts of Amazon. The fruit juice and tea of the bark are very common diarrhoea remedies used by Curanderos and local people throughout the Amazon. The antimicrobial activities of *Anacardium occidentale* extracts have been confirmed[5–8]. Its fruits were shown to exhibit antibacterial activity against *Helicobacter pylori* that is commonly implicated in acute gastritis and stomach ulcers[5]. The antidiabetic and antiinflammatory properties of the leaf and bark extracts of the cashew plant have also been validated[3,4,9]. The observed antiinflammatory and antidiarrhea activities of cashew extracts have been attributed to tannins.

Anacardic acids, one of the phytochemical constituents of cashew extract, has been shown to curb the darkening effect of aging by inhibiting tyrosinase activities and kill certain cancer cells[10]. Mendes[10] has documented a wide range of chemicals isolated and identified from cashew. They included anacardic acids, anacardol, hydroxybenzoic acid, kaempferol, salicylic acid and tannins.

The toxic effects of herbal preparations on target organs of animals and humans are enormous hence have elicited tremendous medical concerns[1,11]. The potential toxic substances in plants such as antinutritional factors, heavy metals and phytochemicals, have been the subject of many investigations[12,13]. Researchers had adopted in the past either physicochemical or biological approach to assessing the toxic potentials of medicinal plants on man and animals. No existing report, to the best of our knowledge, has adopted both approaches to the assessment of medicinal plants toxicity. The use of *Anacardium occidentale* extract in the management of chronic ailments necessitated the assessment of the extract’s hepatopathic effect on chronic administration. Thus we adopted physicochemical and biological approaches in the evaluation of the toxicological profile of the stem bark of *Anacardium occidentale*.

The inner stem bark of *Anacardium occidentale* was collected at Ajuona Primary School, Nsukka, Enugu State, Nigeria, in June 2007 and, identified in the Department of Botany, University of Nigeria, Nsukka. A voucher sample of number UNH/240 was deposited at the departmental herbarium. The inner stem bark was air-dried and pulverised to coarse powder and 0.5 kg of the coarse powder was macerated in 2.5 l of 80 % methanol for 48 h. The solvent was distilled off under vacuum at less than 40^°^to obtain reddish brown solid. The phytochemical constituents of the extract were determined using standard methods[14,15].

Wistar rats and albino mice of either sex weighing between 160-245 g and 18-23 g, respectively, were obtained from the Animal House Unit, Faculty of Vetinary Medicine, University of Nigeria, Nsukka. They were fed freely with standard feed and water before the commencement of the experiment. The experimental animals were handled in accordance with stated guidelines of the University of Nigeria Ethical Committee.

Nine mice divided into three groups of three animals per group were treated orally with three doses (10, 100 and 1000 mg/kg) of the extract, respectively. They were observed over a 24 h period for gross behavioural change and mortality. No mortality was noted; and three more mice were administered 1600, 2900 and 5000 mg/kg doses of the extract, orally, at one dose per animal. Animals treated with 2,900 and 5,000 mg/kg doses died within 48 and 24 h after treatment, respectively. The LD_50_was evaluated according to Lorke’s method[16].

The rats were divided into three groups of four rats per group. They were then put in separate metal cages and fed freely with standard feed and water. The extract, dissolved in distilled water, at 1.44 and 2.87 g/kg were administered per oral daily for two weeks to rats in respective groups. Rats in control group did not receive any treatment for the same period of two weeks. At the end of two weeks, the rats were fasted over night and anaesthetised with chloroform on the 15th day. Blood was collected via heart puncture and placed in dried labelled vials to clot.

The blood collected as described above was centrifuged at 3000 rpm for 10 min to obtain the serum. Liver function analysis for serum alanine aminotransaminase (ALT), aspartate aminotransaminase (AST), alkaline phosphatase (ALP), total bilirubin (TB) and total protein (TP) were performed using standard methods [17–19]. The levels of tannins, saponins, oxalates, phytate, cyanide, iron, lead and cadmium, as antinutrients and heavy metals, in the A. *occidentale* inner stem bark were determined by standard methods as previously described[20]. Results of the liver function indices were expressed as mean±SEM and were compared using student t-test. The obtained results were considered significant at *p*<0.05.

The phytochemical composition of the extract as shown in Table 1 indicated limited amounts of free reducing sugar, moderate amounts of saponins and abundance of tannins. Table 2 presented the levels of anti-nutrients determined in the crude drug. Tannins had the highest concentration per 100 g of sample, on air-dried basis, while low level of cyanide was detected. The anti-nutrients levels ranged from 0.025-5.75 percent. The level of some heavy metals in the stem bark is shown in Table 3. The iron content level was 8.92 mg/100 g, while lead and cadmium were non-detectable.

**TABLE 1:** PHYTOCHEMICAL COMPOSITION OF THE EXTRACT

Phytocompound	Approximate level
Condensed tannins	+++
Hydrolysable tannins	+++
Pseudotannins	+++
Saponins	++
Free reducing sugar	+
Glycosides	++

++++ Excessive; +++ High; ++ Moderate; + Low; – Absent.

**TABLE 2:** ANTINUTRIENTS LEVELS IN *ANACARDIUM OCCIDENTALE* INNER STEM BARK

Antinutrient	Concentration (%)
Saponins	2.000
Oxalate	2.500
Tannins	5.750
Phytate	0.250
Cyanide	0.025

**TABLE 3:** HEAVY METALS LEVELS IN *ANACARDIUM OCCIDENTALE* INNER STEM BARK

Heavy metal	Amount (mg/100 g)
Iron	8.92
Lead	ND
Cadmium	ND

ND is not detectable

The effect of the sub-chronic administration of the extract on liver function parameters of rats was determined and the result presented in Table 4. The extract induced significant increase in the levels of ALT and AST (*p*<0.05). Serum levels of ALP and TP were, also, dose-related but were not significantly (*p*< 0.05) different. TB levels were irregular and either lower than or close to the control. The LD_50_per oral in mice was evaluated to be 2,154.07 mg/kg.

**TABLE 4:** SERUM LEVELS OF LIVER FUNCTION INDICES IN EXTRACT-TREATED AND NORMAL WISTAR RATS

Serum liver function indicator	Treatment I (2.87 mg/kg)	Treatment II (1.44 mg/kg)	Normal Rats
ALT (µ/l)	63.00±11.24*	35.00±4.10*	10.62±3.13
ALP (µ/l)	95.72±9.30	80.35±10.47	96.60±2.80
AST (µ/l)	144.00±32.60*	94.25±1.44*	10.00±3.00
TB (µmol/l)	0.61±0.04	1.65±1.07	1.65±0.16
TP (g/dl)	8.45 ± 0.74	8.08 ± 0.44	7.47 ± 1.05

* Significant at *p*< 0.05, N=4 Wistar rats per group; ALT: alanine aminotransferase. ALP: alkaline phosphatase; AST: aspartate aminotransferase; TB: total bilirubin and TP: total protein.

Saponins possess surfactant and haemolytic properties especially on parenteral administration. When administered orally, saponins are non-toxic becausehydrolytic enzymes in the gastrointestinal tract (GIT) readily hydrolyse it to its component aglycone (sapogenin) and glycone. None of these products, on absorption into the systemic circulation, can cause erythrocyte haemolysis hence saponins are classified as non-toxic if orally administered. Tannins, as polyphenols, chelate metal ions and form irreversible complexes with macromolecules like proteins, carbohydrates and enzymes. They are astringent and toxic to rumen microorganisms like *Streptococcus boris, Butyribrio fibrosolrens* and *Fibrobacter succinogens*. Three mechanisms of tannins toxicity so far identified include: enzyme inhibition and substrate deprivation, action on membranes, and metal ion deprivation. Tannins also induce changes in morphology of several species of luminal bacteria[21,22]. In excessive amount, ingested tannins could depress growth rates, lower protein utilisation, damage the mucosa lining of the digestive tract, alter the excretions of certain cations and increase the excretion of proteins and essential amino acids, in man and other monogastric animals[22]. Oral tannins lower the biological value of dietary proteins, and also, reduce mineral absorption in the GIT. These actions have serious systemic consequences as deficiency of essential amino acids and enzyme co-factors may accompany chronic administration. Tannins toxicity may be evident in the sub-chronic to chronic use of *A. occidentale* stem bark in ethnomedicine.

Oxalate salts, often caused by complexation with metal ions like calcium, magnesium and other di- or trivalent nutrients, lead to the blockage of biological ducts in the kidney and gall as stones[23]. This has occasioned many studies on the correlations of ingestion of plant oxalates on calcium absorption and the possibility of calcium deficiency resulting from oxalate content of foods. Oxalate content of 2.50 per cent is considered toxic and may increase the risk of oxalate stones.

Phytic acid (myo-inositol hexaphosphoric acid) comprises 1-5 percent of most cereals, nuts, legume, oil seeds, pollen and spores[24,25]. It forms insoluble salts with essential minerals like calcium, iron, magnesium and zinc in foods, rendering them unavailable for absorption into the blood stream. About half of the phytic acid phosphorus content is excreted unchanged in man, thus remaining unavailable for utilisation. The action of phytic acid on iron and copper could be beneficial, as both metals are known to catalyse UV photolysis of biologic molecules to produce damaging free radicals. With iron, phytic acid forms a monoferric chelate in which the iron is unavailable for participation in the iron-catalysed formation of hydroxyl radicals through Fenton reaction[24]. Thus, phytic acid is a powerful inhibitor of lipid peroxidation and other oxidative reactions; a property that is especially beneficial in extending seed viability in plants. This could, also, inhibit oxidative damage to cells and tissues in animals. Phytic acid toxicity is not expected as trace amounts (250 mg/100 g) of phytic acid was detected in *A. occidentale* inner stem bark.

Presence of cyanide in plants commonly used as foodstuff and phytomedicine have been reported[26]. In plants, cyanide exists as glycosides or nitriles. The lethal concentration of cyanide, in adult man, has been put at 50-60 mg/kg body weight[27]. However, cyanide concentration of 25 mg/100 g of dried plant material is considered low and not likely to pose any danger. At doses administered, *A. occidentale* bark is unlikely to present any danger of cyanogenic toxicity in man and animals.

Iron is an important mineral in human and animal nutrition. It is necessary for the formation and normal functioning of certain macromolecules like haemoglobin, chlorophyll and haem-proteins. However, iron catalyses the conversion of hydrogen peroxide to hydroxyl radicals in biological systems[24], which promotes lipid peroxidation, cell death and tissue damage. Iron is, also, implicated in the organic mercurials and nickel salts enhanced production of reactive oxygen species (ROS). Thus, excessive amount of iron will favour the development of a pro-oxidative state[28] with serious consequences like lipid peroxidation, DNA damage, carcinogenesis, cell death, tissue damage and aging, involving multiple organs. This may be evident in the chronic administration of *Anacardium occidentale* stem bark preparations. Since, its iron content (8.92 mg/100 g) is appreciably high. Toxic effects associated with lead and cadmium is not expected, as undetectable amounts of these heavy metals were recorded in the crude drug.

The observed lethal dose (LD_50_) of 2,154 0.7 mg/kg, orally in mice, indicated relative safety. From Table 4, serum ALT and AST levels in extract-treated rats at (1.44 and 2.87 g/kg doses) were significantly different from those of the control and may indicate hepatotoxicity. But, the serum levels of ALP, TB and TP in treated animals were statistically same with those of the control (*p*<0.05). The effect of the extract on ALT, ALP, AST and TP were dose-dependent. Against TB, the observed effect was not dose-related; but was either lower than or relatively at par with the corresponding value in the control group.

The findings suggest that the extract of *A. occidentale* was relatively toxic to rat hepatic systems at oral doses of 1.44 and 2.87 g/kg for the period of administration. However, the hepatotoxicity was minimal since defective hepatic metabolism of bilirubin was not observed. The serum levels of TB in treated animals, which were either lower than or close to the control, supported this. Also, TP values did not differ significantly (*p*<0.05) between the control and treated groups.

Hepatotoxicity of herbal medicines has been reported severally[11,29]. Increase in serum enzymes such as ALT, ALP and AST, as well as TP and TB are commonly used as measures of liver injury especially by chemicals[30]. Hepatic damage usually results in increased serum level of bilirubin. High bilirubin concentration causes a complementary increase in serum total protein, particularly albumin. Since these conditions were not evident, even at sub-chronic dosing, with *Anacardium occidentale* stem bark extract, it could be concluded that the extract produced moderate but insignificant degeneration of hepatocytes.
